# Intestinal Exposure to Food-Derived Protease Inhibitors: Digestion Physiology- and Gut Health-Related Effects

**DOI:** 10.3390/healthcare9081002

**Published:** 2021-08-05

**Authors:** Anna Kårlund, Isa Paukkonen, Carlos Gómez-Gallego, Marjukka Kolehmainen

**Affiliations:** 1 Institute of Public Health and Clinical Nutrition, University of Eastern Finland, P.O. Box 1627, FI-70211 Kuopio, Finland; carlos.gomezgallego@uef.fi (C.G.-G.); marjukka.kolehmainen@uef.fi (M.K.); 2 Institute of Biomedicine, University of Eastern Finland, P.O. Box 1627, FI-70211 Kuopio, Finland; isap@student.uef.fi

**Keywords:** protease inhibitors, Bowman-Birk inhibitors, Kunitz-type inhibitors, α-amylase/trypsin inhibitors, trypsin, chymotrypsin, irritable bowel syndrome, inflammation, colorectal cancer

## Abstract

Plant-derived protease inhibitors (PI), such as Bowman-Birk inhibitors and Kunitz-type inhibitors, have been suggested to negatively affect dietary protein digestion by blocking the activity of trypsin and chymotrypsin in the human gastrointestinal system. In addition, some PIs may possess proinflammatory activities. However, there is also scientific evidence on some beneficial effects of PIs, for example, gut-related anti-inflammatory and chemopreventive activities in vitro and in vivo. Some PIs are sensitive to processing and digestion; thus, their survival is an important aspect when considering their positive and negative bioactivities. The aim of this review was to evaluate the relevance of PIs in protein digestion in humans and to discuss the potential of PIs from whole foods and as purified compounds in decreasing symptoms of bowel-related conditions. Based on the reviewed literature, we concluded that while the complex interactions affecting plant protein digestibility and bioavailability remain unclear, PI supplements could be considered for targeted purposes to mitigate inflammation and gastric pain.

## 1. Introduction

The current sustainable development goals encourage the consumption and development of plant-based protein sources [1]. Using plants as protein sources has several beneficial effects on human nutritional status and health; for example, legumes and cereal grains are not only rich in protein, but rich in specific minerals and vitamins, as well as in dietary fiber and bioactive compounds, such as phenolic compounds [2]. One of the challenges in replacing animal proteins with plant proteins is the limiting amount of certain indispensable amino acids. Due to the low level of certain limiting essential amino acids, plant proteins, or specifically, their amino acids, might be poorly bioavailable in the human bodily functions and metabolism [3]. Depending on the source of the plant protein, this may cause problems if a variety of different amino acids are not secured within individual diets. The issue can be overcome by combining legumes, such as common beans or lentils (high in lysine) with cereal grains, such as corn or rice (high in sulfur amino acids), as is often done in traditional food cultures [4,5]. Plant cell structures can also encapsulate plant proteins and other nutrients and thus make them less bioaccessible [6]. Some plant-derived compounds travelling within the plant food matrix may affect digestive processes in the human gastrointestinal (GI) system and affect plant protein bioaccessibility and bioavailability. For example, protease inhibitors (PI) naturally present in legume and cereal grains may affect the nutritional value of foods by inhibiting the action of digestive enzymes on proteins [7].

Proteases are enzymes catalyzing the release of amino acids and small peptides from proteins and peptides, and they are widely expressed and secreted, especially in the human GI tract [8]. Proteases are also found in gut commensals and opportunistic pathogens, while other bacteria produce PIs [9]. In the human GI tract, proteases have essential functions related to protein digestion and catabolism that contribute to key processes in cell growth, tissue arrangement, hormonal signaling, and formation of bioactive peptides [8]. Furthermore, proteases are involved in inflammatory and tumor growth-related metabolism [8]. 

In addition to inhibiting human proteases, legume-derived PIs have been found to cause hypertrophy and hyperplasia of the pancreas and extensive secretion of digestive enzymes in rodents, eventually leading to reduction in growth [10,11]. α-Amylase/trypsin inhibitors (ATI) of wheat, for example, may cause inflammatory responses in sensitive individuals [12]. Interestingly, protease inhibitors have also been successfully used as therapy in several GI diseases when administered orally, for example, in ulcerative colitis (UC) [13]; in addition, they may possess some anticarcinogenic properties [14].

Efficient digestive processes, as well as mucosal protection, are regulated by balancing the proteolytic activities in the lumen and the PI activities on the mucosal surfaces [13]. Even the proteases pepsin, trypsin, and chymotrypsin (enzymes participating in gastric and intestinal food digestion) can damage the lining of the GI tract, in case of the failure of natural protective mechanisms, and contribute to GI inflammation [15]. Colorectal cancer is one of the most common cancers in the Western countries, and many research efforts are now investigating the chemopreventive effects of food-derived PIs against this condition [16,17]. Exposure to plant PIs may thus have both negative and positive effects on human physiology and well-being. 

From the protein digestibility point of view, it has been suggested to be beneficial to destroy the inhibitors by food processing. However, highly intensive processing as such may deteriorate the nutritional quality of protein foods [18], and the potential gut-protective effects of the inhibitors are lost. Many food processing steps may affect plant protein solubility and digestibility, in general. Thus, a high total protein content or even a well-balanced amino acid sequence does not always translate into a good digestibility of a protein. Although sophisticated research methods are currently being used to study protein digestibility and metabolic availability of plant proteins in humans [19,20,21], the nutritional quality of many plant proteins and their processed end products is still rather scantly known.

The aim of this review was to survey the relevance of PIs in gut health and to evaluate their role in plant protein digestibility and bioavailability. We have also discussed whether the focus of the food industry should be on removing or preserving PI activities in foods.

## 2. Plant Protease Inhibitors: Classification and Biological Functions

Plant PIs are usually small water-soluble proteins having many roles in the host biology, and they appear widely in the plant kingdom [22]. Among many other functions in plant physiology, PIs are components of plants’ defensive systems. PIs protect plants against pathogens and also against herbivores; thus, several classes of PIs inhibiting mammal and insect digestive enzymes are often expressed in many plant tissues [22,23]. PIs are present in many common food and feed plants, and PIs from *Fabaceae*, *Poaceae*, and *Solanaceae* have been the most widely studied [24]. In this review, we concentrate on the most common PIs of legumes and cereals, two most important sources of plant protein for human nutrition worldwide [2].

Several reviews have been published on classification, properties, and target proteases of PIs [22,23,24,25]. PIs are classified into families, clans, and subgroups based on their evolutionary backgrounds, protein structures, and catalytic sites. PIs in separate families may share their target proteases, while PIs within one family may inhibit many different proteases. The main PI families present in cereals and/or legumes are the serpin superfamily, Bowman-Birk inhibitor (BBI) family, Kunitz-type inhibitor (KTI) family, potato type 1 inhibitor (PI1) family, and ATI family.

The serpin, or serine protease inhibitor, super family is the largest family of PIs, and its members can be found in numerous plants. Several types of serpins have been extracted from cereal grains, for instance. From the wheat genome, 189 putative serpins have been identified [26]. The serpin super family contains PIs that are active against serine proteases having serine as the nucleophilic amino acid in their active sites. Serpins are protein molecules of 39–43 kDa, and in addition to serine proteases, they may inhibit caspases and some cysteine proteases [23]. 

The most well-known members of the BBI family have been extracted from legumes, such as soybean, pea, lentil, field bean, and chickpea, although BBIs are also found, for example, in wheat, barley, and rice [17]. BBIs form another serine protease inhibitor family, and they usually contain two easily accessible inhibitory domains within their 8–10 kDa molecular structure [17]. 

KTIs are ca. 8–22 kDa molecules that have been found in a wide array of plants, for example, in both legumes and cereals [24]. KTIs form their own family of PIs with homologous sequences [24]. In addition to serine proteases, KTIs may inhibit aspartic and cysteine proteases [23]. There are wide genotypic variations between soybean cultivars in their KTI contents [27]. In addition, significant variation has been found, for example, between grass pea (*Lathyrus sativus* L.) accessions in their BBI and KTI activities [28].

Subtilisin/chymotrypsin inhibitors (SCI) are serine protease inhibitors belonging to the PI1 family. Unlike most of the other PI families, PI1 family members do not contain disulfide bonds [29]. Wheat and barley, for example, possess SCIs, in addition to the *Solanaceae* family. SCIs are water soluble, monomeric molecules of ca. 6–8 kDa [23,29,30]. It has been found that the level of SCIs is regulated in wheat according to the cultivar, showing significant differences between wheat genotypes [31].

Cereal ATIs having PI activity block serine proteases; nevertheless, all ATIs do not have inhibitory activity against trypsin activity but block only α-amylase [23]. ATIs of barley and rye, however, have both inhibitory activities [23]. The molecular weight of ATIs is ca. 13–15 kDa, and their level varies largely between plant cultivars [32]. 

## 3. The Impact of Protease Inhibitors in Food Digestion

The purpose of dietary proteins is to provide essential amino acids to build proteinic substances of the human body: structural and storage components, signaling molecules, and biocatalysts. The nutritional quality of dietary proteins, that is, how bioavailable their amino acids are as building blocks for human proteins, depends on their amino acid content and digestibility and further, the integrity and absorption of released amino acids [18]. Digestibility and assimilation of plant proteins is partially affected by the antinutritional factors that accompany them: for example, tannins and PIs interfere with digestive events by precipitating proteins and binding protein-hydrolyzing enzymes, respectively [7]. 

PIs are known to inhibit digestive enzymes mainly by competitive binding [7,33,34]. This means that they block the active site of proteases by binding to their critical portions, thus preventing the true substrates from binding [33]. Serpins, BBIs, KTIs, SCIs, and ATIs inhibit trypsin and/or chymotrypsin, two serine proteases that are formed in the small intestine from their pancreatic proenzymes [35]. In the human digestive system, approximately 1–2 g of both trypsin and chymotrypsin is secreted daily [36,37].

For example, barley serpin has overlapping reactive sites that may inhibit both trypsin and chymotrypsin [38]. Wheat serpins have inhibitory sites for chymotrypsin [39], while oat serpins may inhibit both trypsin and chymotrypsin, chymotrypsin and elastase, or elastase only [40]. Dicot BBIs (e.g., from Fabales; legumes) bare reactive sites that commonly are specific towards both trypsin and chymotrypsin, as well as elastase [25]. It has been speculated that by ingesting 100 g of uncooked soybean or 200 g of lentils or other legumes per day, it would be possible to inhibit all trypsin and chymotrypsin that is produced in the small intestine [36,37]. However, legumes are most often consumed in cooked, or in some other way processed, form, and the intake of fresh, uncooked legumes (e.g., green peas, green beans) can be expected to be mostly small and seasonal. Monocots (e.g., Poales; cereals) produce two types of BBIs having inhibitory activity for trypsin; however, monocot BBIs apparently have lost the activity towards chymotrypsin during their evolution [25]. KTIs, although sometimes reactive with proteases other than serine proteases, show inhibitory activity for trypsin and chymotrypsin, as well [41]. KTIs with antitrypsin and antichymotrypsin activities can be found in soy and other legumes [42], and KTIs with α-amylase inhibitory activity can be found in cereals [43]. From the human nutrition point of view, the most relevant attribute of SCIs is their inhibitory activity towards mammal chymotrypsin [29,44]. It has been suggested that introducing inactive SCI genes to wheat might be a good option to improve the nutritional quality of the crop [44]. In addition, some buckwheat PI1 inhibitors have been found to inhibit mammal trypsin [45].

PIs have been found to upregulate the secretion of cholecystokinin and, consequently, to upregulate the secretion of trypsin and chymotrypsin. Inhibition of the digestive enzymes leads to accumulation of undigested protein in the small intestine and to slower gastric emptying [33]. This way, PIs may help to regulate hunger and food intake and, thus, to tackle obesity [33]. However, it has been suggested that the inhibition of digestive enzymes by PIs may lead to oversecretion of the digestive enzymes, with potentially adverse outcomes, such as inflammation [13].

Reduction in protein digestibility by PIs may result in more undigested protein ending up in the colon, where they are fermented by gut microbiota. Some end-products of peptide and amino acid fermentation may serve as precursors for short-chained fatty acids (SCFA), such as butyrate and propionate, which have been shown to have beneficial effects on human health; however, SCFAs are usually produced from dietary carbohydrates, and the health effects of amino acid-derived SCFAs are less well known [46]. In addition, potentially harmful derivates of indole and phenol, as well as ammonia, sulfides, and biogenic amines, can be produced from amino acids in the colon [46]. Large amounts of undigested proteins in the colon may modify gut microbiota composition by enhancing the numbers of species capable of protein fermentation. Although a diet rich in plants is considered to have overall beneficial effects on human health and gut microbiota, the sources of amino acids may not be recognized and separated in the colon, and thus, high amounts of undigested proteins are potential contributors of microbial metabolites with still unknown variable systemic and metabolic effects in humans. At least, poorly digested proteins may cause gastric symptoms and discomfort in sensitive individuals [46].

ATIs have gained some attention due to their pro-inflammatory activity and ability to cause non-celiac wheat sensitivity [12,47,48]. Wheat ATIs have been found to provoke inflammatory reactions in the intestine via toll-like receptor 4 (TLR4) [48], and it has been demonstrated that ATIs might be capable of binding directly to the receptor [49] and activating the complex of TLR4, cluster of differentiation 14 (CD14), and myeloid differentiation factor 2 (MD2) [48]; this then results in an innate immunity system activation and release of pro-inflammatory cytokines. Thus, the pro-inflammatory activity of ATIs is not linked with the undigested proteins but with ATIs themselves.

## 4. Protease Inhibitors in Foods

### 4.1. Activity of Legume Protease Inhibitors after Food Processing and Home Cooking

The fact that plant protein foods often go through some level of processing before they are consumed leads to the question of whether PIs have any relevance in human digestion physiology and intestine-related health effects. More specifically, although major protease inhibitory activity stems from KTIs in raw soy milk, for example, different types of treatments, such as boiling, autoclaving, and microwave irradiation, significantly reduce KTI in soy, as well as in other types of beans [27,50,51]. It has been suggested that KTIs can be aggregated during heat treatments, which may cause a loss in inhibitory activity, especially in the presence of sodium chloride [51]. Boiling in an alkaline condition most effectively diminishes KTI activity [50], whereas the efficiency of microwave radiation in inactivating KTIs may be further enhanced if the seeds are soaked [27]. Despite the fact that no heat treatment is needed when processing soybeans by sprouting, this treatment may still also cause a significant reduction of soybean KTI [27]. Meanwhile, active KTIs have been detected in soy flour hydrolysate prepared with pancreatin at 50 °C, as well as in its 0.5–10 kDa ultrafiltered fractions [52]. PI1 family members are, overall, suggested to have good resistance to heat [30]. However, this depends on the plant species, as well as on heating conditions. For example, buckwheat SCIs are highly stable when heated (100 °C for 15–30 min) in acidic conditions (pH 3.1); heating in a higher pH may result in lower activity [30]. Subtilisin-inhibiting activity of an adzuki bean PI can be retained after heating at 80 °C for 10 min (pH 4–10), while 50% of the activity was lost when the PI was heated at 100 °C for 10 min in pH 12 [53]. For a barley SCI, it has been observed that the molecule is stable during malting but not during brewing; this is because the brewing conditions are reducing and involve heating [54]. BBIs, in general, have shown good resistance to processing in many kinds of conditions.

Although BBIs, as well, can be slowly inactivated by heating [17,50,51,55], BBIs show no tendency to form aggregates after heating, possibly due to their highly hydrophilic nature [51]. Instead, BBI peptide bond cleavage may result in inactivation of these inhibitors in soy milk; however, this requires high-energy impact [51]. Other components present in plant-based whole foods may alter the kinetics of BBI activity, and Maillard reaction, for example, may cause some losses in trypsin inhibitory activity (TIA) and/or chymotrypsin inhibitory activity (CIA) [56].

In a molecular-level study of soybean BBIs it was observed that glycation during food preparation may reduce TIA of the BBI isoinhibitor IBBD2 but does not affect TIA in another isoinhibitor IBB1 [16]. It was suggested that although the differences in the amino acid sequence of BBI isoinhibitors are minor, they still expose IBBD2 to a loss of activity when glucose is present during boiling (95 °C, 120 min) [16]. Boiling, as such, did not have any major effects on TIA of IBB1, while TIA of IBBD2 was significantly reduced. CIA of IBB1 was not altered by boiling with or without glucose. No CIA was observed for IBBD2, whatsoever [16]. For chickpeas, it has been observed that soaking (9 h) in citric acid or sodium bicarbonate does not affect total TIA, while cooking (35 min) after the soaking step in water, citric acid, or sodium bicarbonate eliminates TIA [57]. Soaking in water alone or dry heating at 120 °C (15 min) also lowers TIA in chickpeas [57]. These results indicate that both the type of the inhibitors, as well as the processing parameters, determine whether the inhibitory activities are preserved or not. Furthermore, if the inhibitors are to be deactivated, separate inactivation procedures might be required. TIA and CIA in some plant protein sources and their products are presented in Table 1.

### 4.2. Trypsin and Chymotrypsin Activity in Cereals and Their Products

In a comparative study of wheat, rye, and Triticale seeds, rye showed the highest total TIA [59]. However, TIA values showed large variations between harvests and cereal varieties [59]. Wheat flour has been found to have the lowest TIA in comparison to rye mix flour (roasted malt, vital gluten, and bread making additives) and to mixed cereal flour (rye flour, wheat flour, sunflower seeds, flaxseed, sesame, roasted malt, wheat bran malt extract, and bread making additives), which had the highest TIA (Table 1) [58]. When corresponding breads were analyzed, rye bread displayed the highest TIA. Rye flour and wheat bread exhibited the highest CIA [58]. Interestingly, whole wheat flour or bread did not show any TIA [58]. The authors suggested that the bran and its complex polysaccharides interact with whole wheat trypsin inhibitors, thus reducing their activity [58]. Instead, whole wheat flour and bread showed some CIA: in whole wheat flour, CIA was the lowest in comparison to wheat, rye, and mixed flour, while CIA in whole wheat bread was the second lowest after mixed cereal bread [58]. It has been reported that serpins in cereals have variable inhibitory activities towards trypsin and/or chymotrypsin; for example, barley serpins have the ability to inhibit both trypsin and chymotrypsin, while wheat serpins inhibit chymotrypsin and cathepsin G, but not trypsin [23]. Moreover, rye and barley ATIs inhibit trypsin, while similar inhibitors from some other cereals show inhibitory activity towards amylase, only [23]. It has also been suggested that the genetic and geographic origin of the raw material affects ATI levels and activity in their end products [60]. Variability in these activities may thus explain the differences seen in flour and bread types. 

Yeast fermentation and baking caused a significant loss in TIA of mixed cereals. Interestingly, however, a significant increase in CIA of whole wheat was observed after the fermentation and baking steps. Baking did not influence the CIA of rye bread and mixed cereals bread [58]. The effects of fermentation on TIA and CIA of legumes and (pseudo)cereals has recently been reviewed by Kårlund et al. [1]; the general conclusion was that fermentation often reduces protease inhibitory activity. Nevertheless, food processing steps may cause some protein fragments with CIA to be formed or exposed, thus increasing the inhibitory activity [1]. ATIs are also susceptible to degradation by sourdough fermentation, and their pro-inflammatory effects may be diminished in wheat breads that have been fermented with lactic acid bacteria instead of yeast [12].

It must be noted that very variable methods are used to measure TIA and CIA; enzyme inhibition can be presented as weight or enzyme units per amount of product or protein. Furthermore, in some studies, the reduction in TIA or CIA is reported as a percentage. Thus, TIA or CIA results from different studies cannot very often be compared, as such. Furthermore, it is challenging to evaluate PI activity and their capability to inhibit digestive enzymes in humans in vivo based on TIA and CIA observed in foods in vitro. As trypsin and chymotrypsin inhibition is dynamically compensated by an increase in cholecystokinin and trypsin and chymotrypsin secretion, static in vitro systems do not necessarily tell the whole truth about PIs’ effect on protein digestion. More attention should be paid towards TIA and CIA as measures of potentially bioactive compounds reaching the colon and having effects (positive or negative) on epithelial cells and on gut microbiota.

### 4.3. Stability of Food-Derived Protease Inhibitors during In Vitro and In Vivo Digestion 

In vitro digesta of rye bread, as well as in vitro digesta of wheat breads, showed some TIA; this shows that the digestive enzymes and/or other components of digestive fluids are important for the release of PIs from the food matrix [58]. It was found that trypsin inhibitors in wheat bread were stable in the presence of digestive enzymes, although the inhibitors in rye mix bread showed sensitivity to pepsin digestion. In contrast, chymotrypsin inhibitors present in rye bread were not as stable to pepsin digestion as against intestinal digestion. In general, gastric and intestinal digesta of the breads were higher in TIA than in CIA [58]. Adzuki bean subtilisin inhibitor was found to lose activity upon incubation with trypsin and chymotrypsin [53].

In an in vivo pig model, it has been observed that BBIs present in chickpea meal can survive GI digestion in their active form [61]. The initial TIA and CIA in the chickpea meal were 863 ± 120 and 886 ± 99 units per 100 mg, respectively, and it was evaluated that 7.3 and 4.4% of functional BBIs survived the digestion, based on ileal TIA and CIA, respectively [61]. The observations regarding the survival of PIs in the GI system are important when considering their potential clinical effects. 

It can be concluded that many common staple foods contain active PIs, and some of these PIs retain their activity after GI digestion. The relevance of PIs in compromising protein nutrition and gastric comfort is not easy to evaluate, however, as inhibition of digestive proteases is dynamically regulated in the complex human GI system, and it is not precisely known how much PIs are needed to inhibit trypsin and chymotrypsin on a significant level in vivo. Further, plant foods also contain other components affecting protein digestibility. In addition, the PI activities in food plants vary according to many pre-processing aspects, such as plant species, cultivar, growing season, and stress factors, and are susceptible to processing and cooking. Indeed, due to the high sensitivity of KTIs to processing and due to the mostly proinflammatory effects of cereal serpins, the health effects of BBIs have aroused the most interest.

## 5. Gut Health-Related Effects of Food-Derived Inhibitors

### 5.1. Irritable Bowel Syndrome and Ulcerative Colitis

PIs, especially serine PIs, might be applicable for therapeutic use in the treatment of irritable bowel syndrome (IBS), a chronic GI disorder [8,13,62]. Elevated protease activity in the intestinal mucosa or in the stool samples of individuals suffering from IBS is often reported, and it could potentially be used as a diagnostic target for this condition [13,62,63]. Small intestinal mucosa of IBS patients often express the mRNA for the induction of protease trypsinogen IV on enhanced levels [13]. Visceral hypersensitivity, regularly observed in IBS patients, can be related to elevated trypsinogen IV levels and a response to GI inflammation [13]. In general, serine proteases have been suggested to be an important player in visceral hypersensitivity [8]. When activated, inflammatory cells (e.g., mast cells, T-cells, neutrophils) might release large amounts of inflammatory mediators, including proteases that can sensitize peripheral afferent neurons and induce hypersensitivity [8,62].

Luminal and/or parietal proteases may play a role in the development of defects in intestinal permeability, which are also common in IBS [13,63]. Stress can further increase intestinal paracellular permeability (IPP) and, thus, worsen the hypersensitivity related with IBS. In one study, a fermented soy germ product containing BBIs demonstrated protective effects against a stress-induced increase in IPP in female rats when the animals were supplemented 15 days prior to an induced acute stress period of 2 h (Table 2) [63]. The daily dosage of BBI chymotrypsin inhibitory units was 1 per day; however, in addition to BBIs, the rat diets also contained 0.45 mg of potentially bioactive isoflavone aglycones per day per animal [63]. Protease-activated receptors (PAR), regulated by protease molecules, participate in cellular functions and potentially contribute to visceral hypersensitivity (Table 2) [8,62]. PARs can be categorized by four different types: PAR1, PAR2, PAR3, and PAR4; PAR2 has shown the most pivotal role in the pain sensations related to IBS [8,13]. Some studies have suggested that while pancreatic trypsin is involved in the activation of PAR2, trypsin inhibitors might suppress this pathway, thus relieving hypersensitivity [63,64]. Intestinal motility, another pathogenic factor in IBS subtypes (i.e., diarrhea and constipation types), is also suggested as being regulated by PARs [13].

In preclinical studies, BBIs present in soybean have demonstrated some potential in reducing inflammatory responses [17]. It has been suggested that the anti-inflammatory effect is gained by preventing the release of oxygen free radicals from damaged cells [17]. Although the endogenous PIs present in human serum are usually able to balance the protease activity, elevated proteolytic damage on the extracellular matrix can often be seen in an inflamed gut. In the inflammatory state of UC, for example, concentrated BBIs might function as additional inhibitors of excessive protease activity [65]. Indeed, BBIs from soybean might be capable of reducing the activity of elastase, human cathepsin, and human mast cell chymase; these protease enzymes are involved in inflammation and contribute to the disruption of the extracellular matrix (Table 2) [65]. Isolates of trypsin and chymotrypsin inhibitors from the seeds of *Erythrina velutina*, a woody member of the legume family, have also demonstrated elastase inhibiting activity and, further, protective effects on gastric mucosa in a rat model with induced gastric ulcers (Table 2) [66]. The dose of *E. velutina* PIs was 0.2–0.4 mg/kg. 

It would appear that relatively low doses of trypsin, chymotrypsin, and elastase inhibitors would be effective in reducing the symptoms related with IBS [63] and UC [66]. However, accurate doses are rarely reported, while inhibited protease units are more often presented. In a clinical trial on patients with active UC, a dosage of 800 chymotrypsin inhibitory units per day in the form of soy BBI concentrate was shown to be effective in mitigating the disease without any signs of toxicity or other adverse effects [65]. Instead, wheat and its ATIs have shown pathogenic effects in the intestinal tissues of wildtype mice with chemically induced UC when their diets were supplemented with 30% of wheat or with ATIs in equal amount to a standard human diet, i.e., to 150 g of wheat flour per day, in otherwise wheat-free diets (Table 2) [67]. After 2 weeks of supplementation, wheat caused an increase in, for example, the expression of Tumor Necrosis Factor α (TNFα), Interleukin (IL) 17, and IL-6, while both a wheat-based diet and wheat-free ATI-supplemented diet caused body weight loss, shortening of the colon, and histological changes in the intestine [67]. Wheat ATIs have been found to induce IL-8 and monocyte chemoattractant protein 1 (MCP-1) secretion in human myeloid THP1 cells in vitro in a dose-dependent manner (0–500 µg/mL) [60]. In addition, single oral dose of 100 mg/kg of wheat ATIs (corresponding to a human dosage of 8.1 mg/kg or 567 mg/day; typical range in the diet 500–1500 mg/day) caused an increase in transcript levels of IL-8, MCP-1, TNF-α, and IL-15 24 h after the ATI exposure in the distal ileum of mice on a gluten-free diet; serum levels of IL-6 peaked in 5 h (Table 2) [60]. In UC mice fed with a gluten-free diet and exposed to an increasing oral ATI dose (25 mg/kg on days 1, 3, and 6; 100 mg/kg on day 8), transcript levels of IL-8, MCP-1, and IL-6 were increased in the distal ileum within 4 h after the final ATI dose, and serum IL-6 and IL-8 were also increased [60]. However, no intervention studies on ATIs and their effects on human innate immunity responses have been conducted yet [68].

### 5.2. Suppression of Colorectal Cancer

BBIs achieved the Investigational New Drug status already back in 1992, and no toxic or other adverse effects have been observed in humans [7,17,65]. The fact that BBIs with inhibitory activity towards chymotrypsin is maintained during GI digestion in vivo is especially of interest, as it may indicate that this activity is relevant in colon cancer prevention [61]. Based on an in vitro study, the effective concentration of BBIs in the growth medium of human colorectal adenocarcinoma cell line HT29 has been suggested to be greater than 31 µM; some minor differences in this in vitro bioactivity may exist between BBI isotypes IBBD2 and 1BBI [14]. It has also been suggested that depending on agricultural and processing factors, commercial soy milks may contain high enough concentrations of BBIs to be relevant in cancer prevention when consumed regularly [55,56]. However, as the exact anticancer mechanisms and targets of soy BBI isoinhibitors are unknown, the true significance of soy milks in carcinogenic processes is hard to evaluate [55]. The cancer-preventive effects of plant-derived PIs in cell in vitro systems and animal in vivo systems have been extensively and regularly reviewed in the last three decades; however, there is not yet much evidence of the plant PIs’ effects on colorectal cancer development in humans [17,69,70]. 

BBIs and KTIs may also protect other bioactive proteinic components, for example, lunasin, from digestion [71]. Actually, the ability to protect lunasin has been suggested to be the most pivotal attribute of PIs in cancer suppression [72,73]. Lunasin is a bioactive peptide derived from food plants, such as soy, and some cereals [72,74]. Lunasin has chemopreventive effects against colorectal cancer cell lines both in vitro and in vivo. It has been found that active IBB1 helps to preserve at least 34% of lunasin (lunasin:IBB1 ratios 1:2 and 1:1 *w*/*w*) during gastric in vitro digestion; inactive IBB1 protects 28% of lunasin [72]. It was suggested that as a larger molecule, IBB1 encapsulates lunasin even in its inactive form, thus protecting lunasin from pepsin activity. Likewise, it is also possible that some of IBB1 present in the in vitro gastric digesta remained active and therefore demonstrated inhibitory activity against pepsin [72]. Due to its TIA and CIA, active IBB1 reduces lunasin hydrolysis by duodenal enzymes in vitro; this effect has been found to be dose-dependent [72]. When HT29 and Caco2 cells have been exposed to in vitro digesta containing peptides derived from both lunasin and IBB1, their growth was reduced in a dose-dependent manner [72]. Together, these results indicated that the chemopreventive effects of soy foods, for example, can be at least partially explained by the interaction of IBB1 and lunasin.

### 5.3. Impact on Gut Microbiota Population and Metabolic Dynamics

For now, studies investigating PI–gut microbiota interactions are scarce. It is well known that gut microbiota produces both proteases and their inhibitors [9]; these bioactive substances may play a role in gut fermentation of dietary proteins, as well as in the regulation of gut microbiota population and metabolism. Two putative serpins have been found to be expressed by human gut bacterium *Eubacterium sireaum* and to inhibit Human Neutrophil Elastase and Proteinase3 [75]. As both of these enzymes have been associated with inflammatory bowel disease (IBD), the authors speculated that these bacterial PIs might help to tackle the condition [75]. Furthermore, microbial subtilisin has been shown to promote platelet aggregation in vitro [76]; this is of interest as thrombosis and inflammation are also connected in IBD [77]. The role of antisubtilisin inhibitors may thus have some therapeutic potential in this respect [78]. Antimicrobial effects of PIs have been studied to some extent, also regarding some gut health-related species [79]. However, the effects of PIs derived from food on the intestinal microbiota composition and functionality has rarely been studied.

Marín-Manzano et al. [80] investigated the impact of 24-h soy BBI exposure on fecal microbiota composition and growth in vitro. No effects were observed in the microbiota composition when the numbers of lactobacilli, bifidobacteria, bacteroides, coliforms, enterobacteria, clostridia, and total anaerobes were monitored. In addition, Utrilla et al. [81] analyzed the effects of soy BBIs (50 mg/kg/day) on the gut microbiota composition in male mice with induced UC. Minor effects were observed after the exposure of 23 days (2 weeks and 9 days before and after UC induction, respectively): after the BBI exposure, mice with UC showed a slight impact in total bacterial and Bacteroides counts in comparison to non-treated mice with UC. However, better results were gained with pea seed extract (15 g/kg/day) and its albumin fraction (1.5 g/kg/day), which restored the bacterial counts in colitic mice close to the counts in healthy control mice; this was suggested to be partially related with the PIs present in the products (Table 2). All colitic treatment groups (BBI, pea seed extract, albumin fraction) showed anti-inflammatory responses in the intestine.

In mice, an ATI-supplemented diet (equivalent to the amount of ATIs present in daily dosage of 150 g of wheat flour in the human diet) has been found to support the expansion of microbial taxa associated with UC, while limiting the proliferation of some human commensals [67]. When the fecal microbiota of ATI-supplemented UC animals was transplanted to UC mice on a wheat- and ATI-free control diet, the severity of symptoms in the control mice were increased [67]. Dysbiosis was induced by the activation of TLR4, as confirmed by feeding ATI-supplemented diets to Trl4^−^/^−^ mice lacking TLR4: no increase in UC severity was observed in Trl4^−^/^−^ mice with chemically induced UC, and it was concluded that to some extent, TLR4 activation might be regulated by microbial ATI-derived metabolites. Interestingly, some *Lactobacillus* seemed to be able to degrade ATIs [67].

It is important to note that the few studies assessing the impact of PIs on microbial groups are based on culturing methods [80] and qPCR for some families and genus [67,81]. Future studies using molecular methods targeting the whole microbial composition and activity (e.g., metagenomics and metabolomics) may contribute to clarify the effect of PIs in microbiota-host interaction.

**Table 2:** In vivo studies on the potential gut-related health effects of plant protease inhibitors (PI). AF-PSE, albumin fraction of pea seed extract; ant., antagonist; ATI, α-amylase/trypsin inhibitor; BBI, Bowman-Birk inhibitor; CIA, chymotrypsin inhibitory activity; DRG, dorsal root ganglion; DSS, dextran sulfate sodium; EB, 17b-estradiol benzoate; ER, estrogen receptor; IBD, inflammatory bowel disease; IBS, irritable bowel syndrome; IL, interleukin; MPC-1, monocyte chemoattractant protein 1; PAR2, protease-activated receptor 2; PIAQ, protein isolate with antichymotrypsin activity; PIAT, protein isolate with antitrypsin activity; PSE, pea seed extract; rHCl, ranitidine hydrochloride; TIA, trypsin inhibitory activity; TLR4, Troll-like receptor 4; SG, soy germ; UC, ulcerative colitis; WD, wheat-based diet; WFA, wheat-free, ATI-supplemented diet; WFD, wheat-free diet; WT, wildtype C57BL/6 mice.

Method	Treatment Groups	Observation	Conclusion	Ref.
animal trial	Female mice ATI challenge *n* = 6 jejunitis/ileitis *n* = 5 colitis *n* = 5	Ingestion of ATIs induced innate immunity responses in healthy WT mice, as well as in mice with induced inflammation in the small intestine or in the colon: along the intestine, proinflammatory genes (e.g., IL-8, MCP-1) were upregulated, and inflammatory cell populations (e.g., CD11c, CD11b, F4/80+) were increased. Mice were fed with a gluten-free diet.	ATIs induce and promote intestinal inflammation in doses corresponding to a low daily intake in human diet (i.e., 567 mg/day).	[60]
animal trial	Male mice WD *n* = 5 WFA *n* =5 Control WFD *n* = 5 Tlr4^−^/^−^ *n* = 5	In comparison to mice on control diets, the wheat- or ATI-containing diets increased inflammation in intestinal tissues of WT mice with colitis; wheat and ATIs also promoted an increase in colitis-related microbial taxa in the feces of colitic WT mice. ATIs also inhibited proliferation of specific human commensal bacteria. Colitis severity was not affected by the ATI-containing diet in Tlr4^−^/^−^ mice lacking TLR4.	Consumption of wheat or wheat ATIs increases intestinal inflammation in mice with colitis. The proinflammatory and dysbiosis-promoting effects are regulated via TLR4 signaling.	[67]
ex vivo, animal trial	Male mice IBS *n* = 6 healthy *n* = 6 IBS in PAR2^−^/^−^ *n* = 6 saline *n* = 6	DRG neurons of PAR2^−^/^−^ mice exposed to supernatants from IBS patient colon biopsies did not show any increase in calcium mobilization ex vivo. Biopsy supernatants from IBS patients also caused thermal hyperalgesia, allodynia, and abdominal contractions in WT mice but did not cause hyperalgesia or abdominal contractions in PAR2^−^/^−^ mice.	In IBS, proteases are released in excessive amounts, and they can directly stimulate sensory neurons and provoke hypersensitivity via the activation of PAR2.	[62]
animal trial	Male mice PSE *n* = 10 AF-PSE *n* = 10 BBI *n* = 10 DSS *n* = 10 Control *n* = 10	Histological damage and inflammatory markers in the intestine were reduced in PSE, AF-PSE, and BBI-supplemented mice with induced UC. UC promoted a reduction in total bacterial counts, lactobacilli, bifidobacteria, and Bacteroides; in the PSE and AF-PSE groups, the counts were restored close to the counts in healthy control mice, while BBI did not show significant effects in comparison to control UC mice.	PSE and AF-PSE decrease the expression of IBD-related inflammatory markers (e.g., cytokines, TLR, proteins involved in maintaining the epithelial barrier function) in the mouse colon, at least partially due to their BBIs and non-soluble polysaccharides. Because of the presence of these components, PSE is most effective in tackling UC-related dysbiosis.	[81]
animal trial	Female rats SG *n* = 16 SG vehicle *n* = 16 EB *n* = 16 EB vehicle *n* = 16 SG + ER ant. *n* = 8 EB + ER ant. *n* = 8	A daily oral treatment of female rats with a fermented SG ingredient impeded intestinal hyperpermeability and visceral hypersensitivity caused by acute stress. The density of colonic resident mast cells was reduced through estrogen receptor ligand activity, and stress-induced increase in fecal protease activity was prevented by the product.	Over the sexual cycle, IBS-like symptoms are influenced by hormonal changes. Treatment with a fermented SG ingredient demonstrates beneficial effects on stress-induced visceral hypersensitivity and epithelial barrier impairment via a local estrogen-like effect associated with the isoflavones present in the product and by inhibiting proteolytic activity via BBI action.	[63]
animal trial	Female rats PIAT 0.2 mg/kg *n* = 6 PIAT 0.4 mg/kg *n* = 6 PIAQ 0.035 mg/kg *n* = 6 rHCl *n* = 6 saline *n* = 6 sham *n* = 6	Protein isolates (PIAT, PIAQ) from *Erythrina velutina* seeds were shown to protect gastric mucosa and to prevent hemorrhagic lesions, edema, and mucus loss in rats with ethanol-induced ulcers. No toxic effects were observed in liver or kidneys.	*E. velutina* seed protein isolates are potentially gastroprotective natural substances that may be used in therapeutic applications in inflammatory conditions, such as ulcers.	[66]
animal trial	Male pigs BBI test group *n* = 5 Control *n* = 2	BBIs from chickpea-based diets arrive in the terminal ileum. Remaining TIA and CIA in the ileal digesta were 7.3 and 4.4%, respectively.	In the pig, significant amount of active chickpea BBIs reach the large intestine. However, to elucidate the chemopreventive effects of these BBIs in humans, further pharmacological studies are needed.	[61]
clinical trial	Men, women, median age 54 years (23–79 years) BBI *n* = 14 Placebo *n* = 14	Patients with UC showed a slight increase in remission rate and clinical response when receiving soybean-derived BBI concentrate (BBIC), in comparison to the placebo group. BBIC was suggested to have anti-inflammatory activity via an inhibitory effect on serine proteases. No adverse effects were reported.	Although the results did not indicate statistically significant improvements in clinical markers, the benefit of BBIC treatment over the placebo suggests that soybean extracts should be further studied in larger clinical trials to confirm their reducing effects on UC symptoms.	[65]

## 6. Conclusions

Serine protease inhibitor families present in plant foods constitute an intriguing compound group with both therapeutic potential towards bowel-related diseases and potentially proinflammatory and protein nutrition-compromising effects in the human GI system. Especially BBIs from legumes have gained positive attention due to their good stability during food processing and digestion and due to their promising activities as lunasin-protecting agents. Instead, based on animal models, cereal-derived ATIs have been suggested to act as proinflammatory compounds in sensitive individuals. However, in many cases, the positive and negative effects of PIs and other plant components are difficult to separate. In Table 3, we have summarized some interesting and critical points to consider in the discussion on PIs and their roles and forms in human diets. As long as the complex interactions affecting digestibility and bioavailability of plant proteins remain elusive, PI supplements for targeted purposes could be a good option to take advantage of the scientifically proven beneficial effects of these compounds. Daily consumption of plant-based whole foods is always highly advisable to ensure an adequate intake of important fibers, vitamins, minerals, and bioactive compounds; furthermore, appropriate food processing or cooking is needed to ensure the overall safety and palatability of plant protein products, even if the active PIs are consequently lost. However, the potential well-being supporting effects of PI concentrates and isolates should not be neglected, especially in conditions where plant-based whole foods may promote symptoms.

## Figures and Tables

**Table 1:** The effects of different food processes on trypsin inhibitory activity (TIA) and chymotrypsin inhibitory activity (CIA) in soybean and cereal grain flours. MWIR, microwave irradiation; NaHCO₃, sodium bicarbonate; nd, not detected; Ref., references.

Food	TIA	CIA	Ref.
Legumes			
chickpea ^1^	10.43 ± 0.77		[57]
chickpea, soaked, water ^1^	9.20 ± 0.67		[57]
chickpea, soaked in water + cooked ^1^	nd		[57]
chickpea, soaked, citric acid ^1^	10.47 ± 0.46		[57]
chickpea, soaked in citric acid + cooked ^1^	nd		[57]
chickpea, soaked, NaHCO₃ ^1^	10.77 ± 0.75		[57]
chickpea, soaked in NaHCO₃ + cooked ^1^	nd		[57]
chickpea, dry heating ^1^	7.60 ± 0.50		[57]
soybean ^2^	67.2 ± 1.3		[27]
soybean, boiled 5 min ^2^	20.2 ± 0.9		[27]
soybean, boiled 10 min ^2^	8.8 ± 0.5		[27]
soybean, boiled 15 min ^2^	3.2 ± 0.3		[27]
soybean, dried seeds, MWIR 1 min ^2^	24.9 ± 1.2		[27]
soybean, dried seeds, MWIR 2 min ^2^	5.7 ± 0.4		[27]
soybean, soaked seeds, MWIR 1 min ^2^	18.2 ± 0.7		[27]
soybean, soaked seeds, MWIR 2 min ^2^	4.7 ± 0.4		[27]
soybean, sprouting 1 day ^2^	57.0 ± 0.9		[27]
soybean, sprouting 2 days ^2^	37.6 ± 1.3		[27]
soybean, sprouting 3 days ^2^	30.2 ± 0.9		[27]
soybean, sprouting 4 days ^2^	33.7 ± 1.2		[27]
soybean, autoclaved ^2^	nd		[27]
soy milk, commercial ^3^	69–510	28–154	[56]
Cereals			
wheat flour ^4^	5.13 ± 3.1	4.19 ± 0.9	[58]
wheat dough ^4^	2.80 ± 0.6	2.77 ± 1.0	[58]
wheat bread ^4^	28.76 ± 14.2	21.56 ± 2.5	[58]
whole wheat flour ^4^	nd	2.16 ± 0.3	[58]
whole wheat dough ^4^	nd	2.18 ± 0.2	[58]
whole wheat bread ^4^	nd	15.32 ± 4.4	[58]
rye mix flour ^4^	15.85 ± 2.9	9.56 ± 2.2	[58]
rye mix dough ^4^	4.75 ± 1.2	2.02 ± 0.3	[58]
rye mix bread ^4^	46.02 ± 11.9	17.31 ± 5.2	[58]
mixed cereal flour ^4^	26.92 ± 3.2	2.49 ± 0.5	[58]
mixed cereal dough ^4^	7.87 ± 3.5	1.30 ± 0.7	[58]
mixed cereal bread ^4^	28.53 ± 13.5	4.93 ± 2.0	[58]

^1^ reduction in activity of trypsin by 1 unit per mg product (dry weight). ^2^ mg trypsin inhibited per g flour. ^3^ inhibited trypsin/chymotrypsin units per ml soy milk. ^4^ reduction in activity of trypsin/chymotrypsin by 1 trypsin/chymotrypsin unit per mg protein.

**Table 3:** Critical points and future considerations in consuming protease inhibitor-containing foods or supplements. FODMAP, fermentable oligosaccharides, disaccharides, monosaccharides, and polyols; IBS, irritable bowel syndrome; PI, protease inhibitor.

Whole Foods	General Population	IBS
PI activities are measured more often than the absolute concentrations of the inhibitors.	It is difficult to separate the health effects of PIs from other plant components, e.g., phytoestrogens and non-soluble polysaccharides.	Oversecretion of digestive proteases is typically observed in IBS patients.
The content of the inhibitors may be challenging to standardize in whole foods due to the natural fluctuation (e.g., biological stress factors, such as insect pests, may affect content).	Western diets typically contain a high amount of animal proteins. In this context, what is the true impact of plant PIs on protein nutrition, and do they possess enough “power” to carry out health-beneficial effects within a normal diet?	Some components in PI-containing plant foods also contain factors contributing to the symptoms of IBS (FODMAPs). What is the role of poorly digested plant proteins in IBS?
Conclusions		
Most accurate information is available from animal models supplemented with purified inhibitors, but there is a lack of knowledge from human studies in the context of the whole diet.	PI supplements could be ingested separately from protein foods; thus, potentially, there would be less interference with protein digestion, and it would be possible to ensure a high enough amount of active PIs in the colon.	Ingesting PIs as supplements could be beneficial for IBS patients due to the lack of compounds contributing to the symptoms.
